# Urethral Calculus in a Horse

**Published:** 1896-05

**Authors:** L. A. Greiner

**Affiliations:** Indianapolis, Ind.


### URETHRAL CALCULUS IN A HORSE.

By L. A. GREINER, V.S.,
INDIANAPOLIS, IND.

In June, 1895, a horse thirteen years old, very low in flesh, was brought to my infirmary for treatment. The history of the case was obscure, the present owner having had the horse only a few weeks, but while in last owner's possession he was noticed to have difficulty in urinating. The attempts were made frequently and with considerable straining and paddling of the hind legs. Soon noticed the above symptoms, and on examination of the urethra I found a large hard lump (the calculus) about 2½ inches below the anus. On casting the horse and cutting down on the stone it was found to be very firmly imbedded in the mucous membranes and difficult to remove. The roughness of the stone accounted for the trouble. Hemorrhage was considerable, several small arteries having to be ligated before the horse was allowed to get up. The external wound was stitched and dressed antiseptically. It took about four weeks to heal entirely over, and at that time all the urine was escaping through the meatus urinarius. Nothing was done in the way of passing a catheter as this was found to be impracticable, the catheter entering some of the pockets formed by the distended mucous membranes at the seat of the operation.

The calculus was of oxalate of lime species. It was irregularly egg-shaped, and at three of the roughened surfaces firmly adherent to the mucous membrane. The following measurements will give an idea of the size of the calculus: Weight, 6 ounces and 2 drachms; longest diameter, 3¾ inches; shortest diameter, 2⅓ inches; longest circumference, 10 inches; shortest, 7½ inches.

